# Supplementary material for: Involvement of Angiopoietin 2 and vascular endothelial growth factor in uveitis
Source: PLoS One. 2023 Nov 28;18(11):e0294745. doi: 10.1371/journal.pone.0294745 (PMC10683998; doi:10.1371/journal.pone.0294745)
Supplement: S1 Table — (DOCX) [file pone.0294745.s001.docx]

**S1 Table. Protein levels of cytokines in the vitreous sample**

| No. |  | Ang1 (ng/ml) | Ang2  (ng/ml) | VEGFA  (pg/ml) | TNF-α  (pg/ml) | IFN-γ  (pg/ml) | IL-17  (pg/ml) |
| --- | --- | --- | --- | --- | --- | --- | --- |
| 1 | Control | 89.2 | 52.9 | 15.6 | 7.1 | 25.3 | 10.5 |
| 2 | Control | 74.2 | 55.8 | 29.4 | 6.8 | 24.0 | 10.5 |
| 3 | Control | 89.2 | 64.6 | 14.5 | 7.1 | 25.3 | 10.5 |
| 4 | Control | 64.7 | 41.9 | 16.4 | 6.8 | 22.8 | 10.5 |
| 5 | Control | 69.4 | 61.7 | 14.2 | 6.8 | 25.3 | 10.5 |
| 6 | Control | 60.1 | 39.2 | 34.9 | 6.4 | 21.5 | 10.5 |
| 7 | Control | 79.1 | 55.8 | 13.5 | 7.1 | 23.4 | 10.5 |
| 8 | Control | 89.2 | 70.7 | 13.8 | 7.1 | 24.0 | 10.5 |
| 9 | Control | 74.2 | 61.7 | 18.3 | 6.4 | 22.8 | 10.5 |
| 10 | Control | 74.2 | 64.6 | 14.2 | 7.1 | 22.8 | 10.5 |
| 11 | Control | 84.1 | 73.7 | 14.5 | 6.8 | 22.8 | 10.5 |
| 12 | Control | 79.1 | 70.7 | 14.9 | 7.1 | 22.8 | 10.5 |
| 13 | Control | 94.5 | 83.1 | 15.3 | 7.1 | 22.8 | 10.5 |
| 14 | Control | 69.4 | 50.1 | 15.6 | 6.4 | 22.8 | 10.5 |
| 15 | Control | 89.2 | 73.7 | 13.1 | 6.8 | 22.8 | 9.5 |
| 16 | Control | 74.2 | 50.1 | 13.5 | 6.8 | 22.8 | 10.5 |
| 17 | Uveitis | 99.8 | 99.3 | 16.8 | 12.8 | 50.8 | 14.8 |
| 18 | Uveitis | 151.8 | 202.7 | 17.9 | 15.8 | 121.5 | 18.4 |
| 19 | Uveitis | 133.6 | 145.6 | 19.0 | 14.7 | 104.8 | 16.0 |
| 20 | Uveitis | 110.7 | 117.7 | 321.3 | 12.1 | 60.2 | 14.8 |
| 21 | Uveitis | 116.3 | 191.4 | 16.8 | 14.3 | 114.0 | 19.0 |
| 22 | Uveitis | 110.7 | 225.7 | 17.5 | 12.8 | 74.9 | 16.0 |
| 23 | Uveitis | 139.6 | 151.0 | 16.0 | 17.3 | 131.0 | 22.2 |
| 24 | Uveitis | 99.8 | 531.2 | 16.0 | 12.8 | 114.0 | 13.7 |
| 25 | Uveitis | 116.3 | 78.4 | 41.7 | 10.2 | 49.3 | 13.7 |
| 26 | Uveitis | 105.2 | 206.5 | 16.0 | 15.0 | 100.2 | 17.2 |
| 27 | Uveitis | 197.0 | 272.9 | 16.8 | 11.0 | 41.9 | 14.8 |
| 28 | Uveitis | 89.2 | 86.3 | 14.9 | 9.9 | 40.4 | 12.6 |
| 29 | Uveitis | 94.5 | 116.0 | 15.3 | 12.1 | 73.3 | 13.7 |
| 30 | Uveitis | 89.2 | 96.0 | 15.3 | 9.5 | 41.9 | 12.6 |
| 31 | Uveitis | 122.0 | 435.2 | 16.8 | 14.3 | 74.9 | 17.2 |
| 32 | Uveitis | 99.8 | 154.6 | 30.2 | 13.5 | 99.3 | 14.8 |

Ang1; Angiopoietin 1, Ang2; Angiopoietin 2, VEGFA; vascular endothelial growth factor A, TNF-α; tumor necrosis factor-α, IFN-γ; interferon-γ, IL-17; interleukin-17.
