# Supplementary material for: Involvement of Angiopoietin 2 and vascular endothelial growth factor in uveitis
Source: PLoS One. 2023 Nov 28;18(11):e0294745. doi: 10.1371/journal.pone.0294745 (PMC10683998; doi:10.1371/journal.pone.0294745)
Supplement: S2 Table — (DOCX) [file pone.0294745.s002.docx]

**S2 Table. Protein levels of cytokines in the serum sample**

| No. |  | Ang1 (ng/ml) | Ang2  (ng/ml) | VEGFA  (pg/ml) | TNF-α  (pg/ml) | IFN-γ  (pg/ml) | IL-17  (pg/ml) |
| --- | --- | --- | --- | --- | --- | --- | --- |
| 1 | Control | 4.5 | 4.4 | 17.3 | 14.8 | 95.1 | 10.9 |
| 2 | Control | 1.7 | 1.0 | 12.5 | 13.1 | 58.4 | 9.0 |
| 3 | Control | 0.8 | 1.1 | 12.3 | 14.1 | 68.8 | 9.9 |
| 4 | Control | 2.0 | 2.9 | 18.3 | 21.6 | 103.5 | 12.2 |
| 5 | Control | 3.2 | 2.6 | 17.3 | 15.2 | 74.0 | 11.3 |
| 6 | Control | 3.4 | 0.8 | 14.3 | 12.7 | 62.5 | 9.9 |
| 7 | Control | 0.95 | 2.1 | 11.8 | 15.6 | 71.9 | 9.9 |
| 8 | Control | 3.7 | 0.9 | 11.8 | 14.1 | 67.7 | 10.4 |
| 9 | Sar | 9.7 | 1.7 | 36.9 | 22.0 | 103.5 | 13.7 |
| 10 | Sar | 2.1 | 4.5 | 13.3 | 16.1 | 71.9 | 10.4 |
| 11 | Sar | 15.1 | 3.5 | 31.2 | 24.1 | 153.4 | 14.1 |
| 12 | Sar | 1.1 | 1.9 | 11.8 | 13.4 | 69.8 | 10.9 |
| 13 | Sar | 1.3 | 2.6 | 31.2 | 20.9 | 113.1 | 13.2 |
| 14 | Sar | 3.9 | 0.9 | 13.8 | 14.8 | 74.0 | 10.9 |
| 15 | Sar | 4.7 | 1.1 | 22.8 | 14.8 | 71.9 | 10.4 |
| 16 | Sar | 10.7 | 2.1 | 35.5 | 22.9 | 103.5 | 12.7 |
| 17 | VKH | 1.3 | 3.3 | 19.3 | 22.0 | 75.0 | 12.2 |
| 18 | VKH | 10.8 | 1.2 | 34.1 | 21.6 | 92.9 | 13.7 |
| 19 | VKH | 27.4 | 1.9 | 16.3 | 17.5 | 78.2 | 11.3 |
| 20 | VKH | 2.1 | 0.6 | 11.2 | 17.0 | 72.9 | 10.4 |
| 21 | VKH | 1.6 | 1.5 | 10.7 | 10.4 | 65.6 | 10.4 |
| 22 | VKH | 18.0 | 1.6 | 49.8 | 12.5 | 77.1 | 18.8 |
| 23 | VKH | 11.2 | 1.4 | 18.3 | 16.5 | 114.2 | 14.1 |
| 24 | VKH | 11.8 | 1.8 | 26.3 | 17.0 | 102.5 | 12.7 |
| 25 | BD | 0.5 | 1.1 | 16.3 | 12.2 | 66.7 | 9.9 |
| 26 | BD | 5.0 | 1.3 | 31.2 | 15.2 | 82.4 | 11.3 |
| 27 | BD | 2.8 | 1.3 | 13.3 | 11.5 | 72.0 | 10.4 |
| 28 | BD | 5.0 | 2.7 | 14.3 | 18.6 | 98.2 | 10.9 |
| 29 | BD | 13.1 | 1.7 | 13.8 | 15.2 | 127.6 | 13.7 |
| 30 | BD | 5.0 | 0.9 | 12.3 | 15.9 | 92.9 | 11.3 |
| 31 | BD | 4.3 | 1.8 | 23.0 | 20.7 | 122.7 | 12.7 |
| 32 | BD | 2.2 | 1.6 | 11.2 | 13.8 | 69.3 | 10.4 |
| 33 | AAU | 6.1 | 1.1 | 16.8 | 20.5 | 105.7 | 13.7 |
| 34 | AAU | 4.3 | 1.3 | 14.8 | 16.8 | 95.1 | 11.5 |
| 35 | AAU | 3.6 | 1.3 | 15.3 | 11.7 | 66.7 | 10.4 |
| 36 | AAU | 4.2 | 2.7 | 15.8 | 17.0 | 101.4 | 11.8 |
| 37 | AAU | 4.5 | 1.7 | 13.3 | 14.1 | 64.6 | 9.9 |
| 38 | AAU | 5.0 | 0.9 | 12.8 | 15.19 | 76.1 | 11.3 |
| 38 | AAU | 8.5 | 1.8 | 30.7 | 17.72 | 87.7 | 11.8 |
| 40 | AAU | 12.1 | 1.6 | 22.8 | 19.47 | 108.9 | 13.2 |

Ang1; Angiopoietin 1, Ang2; Angiopoietin 2, VEGFA; vascular endothelial growth factor A, TNF-α; tumor necrosis factor-α, IFN-γ; interferon-γ, IL-17; interleukin-17. Sarcoidosis; Sar, Vogt-Koyanagi-Harada disease; VKH, Behçet’s disease; BD, HLA-B27-positive acute anterior uveitis; AAU.
