# Supplementary material for: Involvement of Angiopoietin 2 and vascular endothelial growth factor in uveitis
Source: PLoS One. 2023 Nov 28;18(11):e0294745. doi: 10.1371/journal.pone.0294745 (PMC10683998; doi:10.1371/journal.pone.0294745)
Supplement: S3 Table — (DOCX) [file pone.0294745.s003.docx]

**S3 Table. mRNA levels of cytokines in the EAU mice**

|  |  |  | Ang1 |  |  |  | Ang2 |  |  |  | VEGFA |  |
| --- | --- | --- | --- | --- | --- | --- | --- | --- | --- | --- | --- | --- |
|  |  | Day11 | Day16 | Day21 |  | Day11 | Day16 | Day21 |  | Day11 | Day16 | Day21 |
| 1 | Control | 0.004 | 0.689 | 0.998 |  | 0.174 | 0.909 | 1.095 |  | 1.789 | 1.616 | 0.956 |
| 2 | Control | 0.534 | 1.620 | 1.101 |  | 1.957 | 1.344 | 1.838 |  | 0.002 | 0.462 | 0.831 |
| 3 | Control | 1.002 | 0.600 | 0.950 |  | 0.818 | 0.602 | 1.282 |  | 0.305 | 0.171 | 0.475 |
| 4 | Control | 2.460 | 0.424 | 0.764 |  | 1.131 | 0.677 | 0.615 |  | 0.280 | 1.198 | 0.597 |
| 5 | Control | NA | 1.322 | 1.722 |  | 0.887 | 1.372 | 0.632 |  | 2.082 | 0.506 | 1.537 |
| 6 | Control | NA | 1.345 | 0.464 |  | 1.034 | 1.096 | 0.538 |  | 1.542 | 2.047 | 1.603 |
| 7 | EAU | 1.047 | 1.823 | 0.919 |  | 1.358 | 2.181 | 0.658 |  | 1.205 | NA | 1.452 |
| 8 | EAU | 2.021 | 2.347 | 0.619 |  | 0.723 | 2.736 | 0.313 |  | 15.062 | 1.661 | 0.943 |
| 9 | EAU | 0.919 | 2.828 | 1.093 |  | 0.856 | 3.351 | 0.531 |  | 9.893 | 7.498 | 1.072 |
| 10 | EAU | 1.616 | 1.600 | 1.455 |  | NA | 2.850 | 0.747 |  | 4.530 | 10.559 | 1.646 |
| 11 | EAU | 2.736 | 1.281 | 1.089 |  | 0.953 | 3.857 | 0.982 |  | 18.521 | 3.421 | 1.062 |
| 12 | EAU | NA | 1.340 | NA |  | 0.619 | 2.805 | NA |  | NA | 10.396 | NA |

Ang1; Angiopoietin 1, Ang2; Angiopoietin 2, VEGFA; vascular endothelial growth factor A, NA; not available.
