# Supplementary material for: Involvement of Angiopoietin 2 and vascular endothelial growth factor in uveitis
Source: PLoS One. 2023 Nov 28;18(11):e0294745. doi: 10.1371/journal.pone.0294745 (PMC10683998; doi:10.1371/journal.pone.0294745)
Supplement: S4 Table — (DOCX) [file pone.0294745.s004.docx]

**S4 Table. Clinical scores of EAU mice**

|  |  | **Day7** | **Day10** | **Day14** | **Day17** | **Day21** |
| --- | --- | --- | --- | --- | --- | --- |
| **Control IgG Ab** | 1 | 0 | 1 | 2 | 2 | 4 |
|  | 2 | 1 | 1 | 2 | 3 | 4 |
|  | 3 | 1 | 1 | 2 | 2 | 3 |
|  | 4 | 1 | 1 | 2 | 3 | 3 |
|  | 5 | 1 | 1 | NA | NA | NA |
|  | 6 | 0 | 1 | NA | NA | NA |
|  | 7 | 1 | 1 | 1 | 2 | 4 |
|  | 8 | 0 | 1 | 3 | 3 | 4 |
|  | 9 | 1 | 1 | 2 | 3 | 3 |
|  | 10 | 0 | 1 | 2 | 3 | 3 |
|  | 11 | 1 | 1 | 1 | 2 | 3 |
|  | 12 | 1 | 1 | 2 | 3 | 4 |
|  | 13 | 0 | 0 | 2 | 2 | 3 |
|  | 14 | 0 | 1 | 2 | 2 | 4 |
|  | 15 | 0 | 1 | 2 | 2 | 3 |
|  | 16 | 1 | 1 | 2 | 2 | 3 |
|  | 17 | 1 | 1 | 2 | 2 | 3 |
|  | 18 | 1 | 1 | 2 | 2 | 3 |
|  | 19 | 0 | 1 | 1 | 1 | 3 |
|  | 20 | 0 | 1 | 1 | 2 | 3 |
| **Anti-VEGFA Ab** | 1 | 0 | 0 | 2 | 2 | 3 |
|  | 2 | 0 | 1 | 2 | 2 | 3 |
|  | 3 | 1 | 1 | 2 | 2 | 3 |
|  | 4 | 0 | 0 | 2 | 2 | 3 |
|  | 5 | 1 | 1 | 2 | 3 | 4 |
|  | 6 | 1 | 1 | 2 | 3 | 4 |
|  | 7 | 1 | 1 | 2 | 2 | 3 |
|  | 8 | 0 | 0 | 1 | 1 | 2 |
|  | 9 | 0 | 1 | 1 | 1 | 3 |
|  | 10 | 1 | 1 | 2 | 2 | 2 |
|  | 11 | 0 | 0 | 2 | NA | NA |
|  | 12 | 1 | 1 | 2 | NA | NA |
|  | 13 | 1 | 1 | 2 | 2 | 2 |
|  | 14 | 0 | 1 | 1 | 3 | 3 |
|  | 15 | 1 | 1 | 2 | 3 | 3 |
|  | 16 | 1 | 1 | 1 | 2 | 3 |
|  | 17 | 0 | 1 | 1 | 1 | 2 |
|  | 18 | 0 | 0 | 2 | 2 | 3 |
|  | 19 | 0 | 0 | 2 | 3 | NA |
|  | 20 | 1 | 1 | 1 | 2 | NA |
| **Anti-Ang2 Ab** | 1 | 1 | 1 | 1 | 2 | 3 |
|  | 2 | 0 | 0 | 1 | 1 | 2 |
|  | 3 | 0 | 1 | 1 | 2 | 1 |
|  | 4 | 1 | 1 | 1 | 1 | 2 |
|  | 5 | 1 | 1 | 1 | 2 | 3 |
|  | 6 | 0 | 0 | 1 | 2 | 3 |
|  | 7 | 1 | 1 | 1 | 1 | 4 |
|  | 8 | 0 | 0 | 1 | 2 | 4 |
|  | 9 | 0 | 1 | 1 | 2 | 2 |
|  | 10 | 1 | 1 | 1 | 1 | 3 |
|  | 11 | 0 | 1 | 2 | 2 | 4 |
|  | 12 | 0 | 0 | 2 | 3 | 3 |
|  | 13 | 1 | 1 | 2 | 2 | 3 |
|  | 14 | 1 | 1 | 2 | 2 | 3 |
|  | 15 | 0 | 0 | 1 | 2 | 2 |
|  | 16 | 0 | 1 | 2 | 2 | 2 |
|  | 17 | 1 | NA | NA | NA | NA |
|  | 18 | 1 | NA | NA | NA | NA |
|  | 19 | 1 | 1 | 2 | 2 | 2 |
|  | 20 | 1 | 1 | 1 | 2 | 3 |
| **Anti-Ang2 Ab + Anti-VEGFA Ab** | 1 | 0 | 0 | 2 | 1 | 2 |
|  | 2 | 0 | 0 | 2 | 1 | 2 |
|  | 3 | 1 | 1 | 1 | 2 | 2 |
|  | 4 | 0 | 1 | 1 | 2 | 2 |
|  | 5 | 0 | 1 | 1 | 2 | 2 |
|  | 6 | 1 | 1 | 2 | 2 | 2 |
|  | 7 | NA | NA | NA | NA | NA |
|  | 8 | NA | NA | NA | NA | NA |
|  | 9 | 1 | 1 | 1 | 2 | 2 |
|  | 10 | 0 | 0 | 1 | 1 | 1 |
|  | 11 | 0 | 1 | 1 | 1 | 2 |
|  | 12 | 0 | 1 | 2 | 2 | 3 |
|  | 13 | 0 | 0 | 1 | NA | NA |
|  | 14 | 1 | 1 | 2 | 1 | 3 |
|  | 15 | 1 | 1 | 1 | 1 | 3 |
|  | 16 | 1 | 1 | 2 | 2 | NA |
|  | 17 | 1 | 1 | 2 | 2 | 3 |
|  | 18 | 0 | 0 | 1 | 1 | 2 |
|  | 19 | 1 | 1 | 2 | 2 | 2 |
|  | 20 | 1 | 1 | 1 | 1 | 3 |
| **Anti-Ang2/VEGFA bispecific Ab** | 1 | 1 | 1 | 1 | 1 | 2 |
|  | 2 | 1 | 1 | 2 | 1 | 1 |
|  | 3 | 0 | 1 | 1 | 1 | 2 |
|  | 4 | 0 | 0 | 2 | 2 | 2 |
|  | 5 | 0 | 0 | 1 | 1 | 3 |
|  | 6 | 1 | 1 | 1 | 1 | 3 |
|  | 7 | 0 | 1 | 1 | 2 | 3 |
|  | 8 | 1 | 1 | 1 | 2 | 3 |
|  | 9 | 0 | 1 | 1 | 1 | 2 |
|  | 10 | 1 | 1 | 2 | 2 | 1 |
|  | 11 | 0 | 1 | 1 | 2 | 4 |
|  | 12 | 1 | 1 | 1 | 1 | 3 |
|  | 13 | 0 | 1 | 1 | 1 | 2 |
|  | 14 | 1 | 1 | 1 | 2 | 3 |
|  | 15 | 0 | 0 | 1 | 1 | 3 |
|  | 16 | 1 | 1 | 2 | 2 | 2 |
|  | 17 | 0 | 0 | 2 | NA | NA |
|  | 18 | 1 | 1 | 1 | 1 | 2 |
|  | 19 | 1 | 1 | 2 | 2 | 2 |
|  | 20 | 0 | 0 | 1 | 1 | 3 |

NA; not available.
