# Supplementary material for: Involvement of Angiopoietin 2 and vascular endothelial growth factor in uveitis
Source: PLoS One. 2023 Nov 28;18(11):e0294745. doi: 10.1371/journal.pone.0294745 (PMC10683998; doi:10.1371/journal.pone.0294745)
Supplement: S5 Table — (DOCX) [file pone.0294745.s005.docx]

**S5 Table. Histopathological scores of EAU mice**

|  | **Control IgG Ab** | **Anti-VEGFA Ab** | **Anti-Ang2 Ab** | **Anti-Ang2 Ab + Anti-VEGFA Ab** | **Anti-Ang2/VEGFA bispecific Ab** |
| --- | --- | --- | --- | --- | --- |
| 1 | 0.5 | 0.5 | 0 | 0.5 | 0 |
| 2 | 0.5 | 1 | 0.5 | 0 | 0 |
| 3 | 1 | 0.5 | 1 | 0.5 | 0 |
| 4 | 1 | 3 | 1 | 0.5 | 0 |
| 5 | NA | 2 | 0.5 | 0 | 2 |
| 6 | NA | 0.5 | 1 | 0.5 | 1 |
| 7 | 2 | 0 | 1 | NA | 0 |
| 8 | 3 | 0 | 0.5 | NA | 0 |
| 9 | 0.5 | 0.5 | 1 | 0.5 | 2 |
| 10 | 0.5 | 1 | 2 | 1 | 0 |
| 11 | 0.5 | NA | 2 | 1 | 0 |
| 12 | 1 | NA | 0.5 | 0 | 0 |
| 13 | 2 | 2 | 0 | NA | 1 |
| 14 | 2 | 1 | 0 | 0.5 | 0.5 |
| 15 | 1 | 0.5 | 0.5 | 1 | 0 |
| 16 | 2 | 1 | 1 | NA | 0 |
| 17 | 2 | 0.5 | NA | 0.5 | NA |
| 18 | 2 | 1 | NA | 0.5 | NA |
| 19 | 1 | NA | 0.5 | 0 | 0.5 |
| 20 | 2 | NA | 1 | 1 | 0 |

NA; not available.
