# Supplementary material for: Involvement of Angiopoietin 2 and vascular endothelial growth factor in uveitis
Source: PLoS One. 2023 Nov 28;18(11):e0294745. doi: 10.1371/journal.pone.0294745 (PMC10683998; doi:10.1371/journal.pone.0294745)
Supplement: Attachment — Submitted filename: Response to Reviewers.docx [file pone.0294745.s006.docx]

Oct 06, 2023

Koichi Nishitsuka

Academic Editor

PLOS ONE

Dear Prof. Nishitsuka,

We appreciate your careful review of our manuscript and the guidance provided for revision. As requested, we have revised the manuscript, which contains significant corrections according to the guidance provided. The changes made have been highlighted using the "track changes" function of Microsoft Word. Please find below our point-by-point responses to the reviewers’ comments reproduced in *italics*.

Journal Requirements:

When submitting your revision, we need you to address these additional requirements.

1. Please ensure that your manuscript meets PLOS ONE's style requirements, including those for file naming.

*We apologize that we did not describe the manuscript according to the rules of your journal style. We have changed the format of our manuscript.*

2. To comply with PLOS ONE submissions requirements, in your Methods section, please provide additional information regarding the experiments involving animals and ensure you have included details on (1) methods of sacrifice, (2) methods of anesthesia and/or analgesia, and (3) efforts to alleviate suffering.

*We have added the following sentences as the details on methods of sacrifice and anesthesia.*

*“The EAU and control mice were euthanized with an overdose of anesthetia by intraperitoneal injection of Pentobarbital (0.5ml, 1.2%), and their retinochoroidal tissues were extracted on Days 11, 16, and 21 after immunization.” (Page 7, line 134-136)*

*“The clinical score of EAU was evaluated by funduscopy every 3 or 4 days starting from 7 days after immunization under anesthesia by intraperitoneal injection of Pentobarbital (0.1ml, 1.2%).”(Page7, line 152-154)*

*“On Day 21 after the induction of EAU, the mice were euthanized with an overdose of anesthesia by intraperitoneal injection of Pentobarbital (0.5ml, 1.2%).” (Page8, line 158-159)*

3. We note that the grant information you provided in the ‘Funding Information’ and ‘Financial Disclosure’ sections do not match.  When you resubmit, please ensure that you provide the correct grant numbers for the awards you received for your study in the ‘Funding Information’ section.

*We apologize that we did not describe the manuscript according to the rules of your journal style.* *We have removed the conflict of interest statement from the manuscript and have listed declaration of competing interest and financial support as follows.*

*“Competing Interests Statement: RF is an employee of F. Hoffmann-La Roche Ltd. F.Hoffmann-La Roche provided support in the form of salary for the organisation’s author [RF], and he had a role in elements of the study design, and preparation of the manuscript, but no additional roles in the data collection and analysis and decision to publish the manuscript. The specific roles of authors are articulated in the ‘author contributions’ section. DI awarded Grants-in-Aid for Scientific Research by Japanese Society for the Promotion of Science [grant number 16K29294] and a grant of research by AMO Japan (Johnson & Johnson surgical vision) [grant number AS2021A000044497]. This does not alter our adherence to PLOS ONE policies on sharing data and materials.”*

*“Funding Statement: This work was supported by Scientific Research by Japanese Society for the Promotion of Science [grant number 16K29294] and a grants of research by AMO Japan (Johnson & Johnson surgical vision) [grant number AS2021A000044497]. The funders had no role in study design, data collection and analysis, decision to publish, or preparation of the manuscript. F. Hoffmann-La Roche Ltd. provided the mouse cross-reactive tool antibodies used in the experimental autoimmune uveoretinitis experiments described here. F. Hoffmann-La Roche Ltd provided support in the form of salaries for their organisation’s author [RF], and he was involved in elements of the study design, and preparation of the manuscript. The specific role of this author is articulated in the ‘author contributions’ section.”*

4. Please state what role the funders took in the study.  If the funders had no role, please state: ""The funders had no role in study design, data collection and analysis, decision to publish, or preparation of the manuscript."" If this statement is not correct you must amend it as needed. Please include this amended Role of Funder statement in your cover letter; we will change the online submission form on your behalf.

*We have added the sentence of the funders statement in the Competing Interests Statement as above.*

5. Please expand the acronym “JSPS, J&J” (as indicated in your financial disclosure) so that it states the name of your funders in full. This information should be included in your cover letter; we will change the online submission form on your behalf.

*We have added the name of the funders in Competing Interests Statement and Funding Statement as above.*

6. Thank you for stating the following in the Competing Interests section: "The authors declare no competing interests except for RF who is an employee of F. Hoffmann-La Roche Ltd." We note that one or more of the authors are employed by a commercial company: Hoffmann-La Roche Ltd.

(1) Please provide an amended Funding Statement declaring this commercial affiliation, as well as a statement regarding the Role of Funders in your study. If the funding organization did not play a role in the study design, data collection and analysis, decision to publish, or preparation of the manuscript and only provided financial support in the form of authors' salaries and/or research materials, please review your statements relating to the author contributions, and ensure you have specifically and accurately indicated the role(s) that these authors had in your study. You can update author roles in the Author Contributions section of the online submission form.

Please also include the following statement within your amended Funding Statement. 
“The funder provided support in the form of salaries for authors [insert relevant initials], but did not have any additional role in the study design, data collection and analysis, decision to publish, or preparation of the manuscript. The specific roles of these authors are articulated in the ‘author contributions’ section.”
If your commercial affiliation did play a role in your study, please state and explain this role within your updated Funding Statement.

(2) Please also provide an updated Competing Interests Statement declaring this commercial affiliation along with any other relevant declarations relating to employment, consultancy, patents, products in development, or marketed products, etc.

Within your Competing Interests Statement, please confirm that this commercial affiliation does not alter your adherence to all PLOS ONE policies on sharing data and materials by including the following statement: ""This does not alter our adherence to PLOS ONE policies on sharing data and materials.” (as detailed online in our guide for authors <http://journals.plos.org/plosone/s/competing-interests>).

If this adherence statement is not accurate and there are restrictions on sharing of data and/or materials, please state these. Please note that we cannot proceed with consideration of your article until this information has been declared.

Please include both an updated Funding Statement and Competing Interests Statement in your cover letter. We will change the online submission form on your behalf.

*We have added the statement in Funding Statement and Competing Interests Statement as above.*

7.  In your Data Availability statement, you have not specified where the minimal data set underlying the results described in your manuscript can be found. PLOS defines a study's minimal data set as the underlying data used to reach the conclusions drawn in the manuscript and any additional data required to replicate the reported study findings in their entirety. All PLOS journals require that the minimal data set be made fully available. For more information about our data policy, please see <http://journals.plos.org/plosone/s/data-availability>.

Upon re-submitting your revised manuscript, please upload your study’s minimal underlying data set as either Supporting Information files or to a stable, public repository and include the relevant URLs, DOIs, or accession numbers within your revised cover letter. For a list of acceptable repositories, please see <http://journals.plos.org/plosone/s/data-availability#loc-recommended-repositories>. Any potentially identifying patient information must be fully anonymized.

Important: If there are ethical or legal restrictions to sharing your data publicly, please explain these restrictions in detail. Please see our guidelines for more information on what we consider unacceptable restrictions to publicly sharing data: <http://journals.plos.org/plosone/s/data-availability#loc-unacceptable-data-access-restrictions>. Note that it is not acceptable for the authors to be the sole named individuals responsible for ensuring data access. We will update your Data Availability statement to reflect the information you provide in your cover letter.

*We have added the data sets as supplemental tables in supporting information as follows.*

*Supporting information (Page23, line 567-573)*

*S1 Table. Protein levels of cytokines in the vitreous sample (Page25)*

*S2 Table. Protein levels of cytokines in the serum sample (Page26)*

*S3 Table. mRNA levels of cytokines in the EAU mice (Page27)*

*S4 Table. Clinical scores of EAU mice (Page28-29)*

*S5 Table. Histopathological scores of EAU mice (Page30)*

8. We note that you have stated that you will provide repository information for your data at acceptance. Should your manuscript be accepted for publication, we will hold it until you provide the relevant accession numbers or DOIs necessary to access your data. If you wish to make changes to your Data Availability statement, please describe these changes in your cover letter and we will update your Data Availability statement to reflect the information you provide.

*We agreed with your policy.*

9. Please include captions for your Supporting Information files at the end of your manuscript, and update any in-text citations to match accordingly. Please see our Supporting Information guidelines for more information: <http://journals.plos.org/plosone/s/supporting-information>.

*We have added the data sets as supramental tables in supporting information as above.*

10. Please review your reference list to ensure that it is complete and correct. If you have cited papers that have been retracted, please include the rationale for doing so in the manuscript text, or remove these references and replace them with relevant current references. Any changes to the reference list should be mentioned in the rebuttal letter that accompanies your revised manuscript. If you need to cite a retracted article, indicate the article’s retracted status in the References list and also include a citation and full reference for the retraction notice.

*We have verified the references, and changed the reference no. 10; Regula, et al. 2016, to Foxton, et al.2019 (Page17, line 415-418).*

Reviewers' comments:

Reviewer #1：

 The authors detected the expression level of some cytokines, such as Ang-1, Ang-2, VEGFA in the vitreous samples and serum. Also, they investigated the effect of anti-ang2 antibody and anti-VEGFA antibody on the EAU and they suggested that ang-2 and VEGFA might be involved in the uveitis. However, the authors should provide sufficient evidences to support the conclusion. First, the expression level of Ang-2 and VEGFA of the vitreous and serum is not consistent with serum. The expression level of ang-2 and VEGFA in vitreous samples are higher however is no different in serum. So why did the authors choose the Ang-2 and VEGFA as targets for treatment?

*Thank you for your comments. In the previous reports, which we referred to in the manuscript (Page13-14, line305-333), these molecules were shown to be involved in the formation of inflammatory pathologies. As you mentioned, the changes of expression level of these molecules are different between serum and vitreous. Based on this result that the protein levels of Ang2 and VEGFA are high not systemically, but only locally in the ocular tissue with uveitis, we hypothesized that these molecules were mainly involved in the pathogenesis of local ocular inflammation in the uveitis patients. Therefore, we evaluated the possibility of these molecules as therapeutic targets in this study. To clarify this point, we have added the following sentences to the discussion section.*

*“In this study, the protein levels of both Ang2 and VEGFA are high not systemically but locally in the ocular tissue. This result suggested that the main focus of inflammation was in ocular tissue and these molecules were involved in the pathogenesis of ocular inflammation.” (Page12, line287-289)*

In the treatment section of EAU, the pictures of clinical findings and the histopathological figures of the anti-ang2-treated eye balls and anti-VEGFA-treated eye balls should be provided.

*Thank you for your comments. To clarify, clinical scoring was carried out by live fundoscopy, and fundus photographs were not taken and evaluated in this study. So there are no fundus photographs available to add to the manuscript. However, we have added the histopathological figure of anti-Ang2 Ab treated group and anti-VEGFA Ab treated group in Fig 5. We have changed the figure legends as follows.*

*“(B) Histopathology of EAU mouse treated with control IgG Ab results in cell infiltration, vasculitis and granulomas in choroid and retina, and these findings were reduced in EAU mouse treated with anti-VEGFA Ab or anti-Ang2 Ab. That of EAU mouse treated with anti-Ang2/VEGFA bispecific Ab resulted in mild vasculitis, no retinal folds and no retinal detachments.” (Page33, line 68-72)*

In addition, the scientific meaning in the study was unclearly described in the abstract.

*Thank you for your comments. We have added the following sentence as conclusions in the abstract.*

*“Based on these results, VEGFA and Ang2 were shown to be upregulated locally in the eye of both uveitis patients and models of uveitis.” (Page2, line 22-23)*

Reviewer #2:

The study has novel findings about a possible role of Ang2 and VEGFA in the process of uveitis. The findings may lead to new opinions on treatment as they clearly have stated. They have found an increased level of expression/concentration of these agents. These may revolutionize pathophysiological concepts.

*Thank you for your comments.*

I have one concern: to obtain vitreous samples from 16 patients with idiopathic uveitis, the authors have performed pars plana vitrectomy -an aggressive procedure with potential complications- in cases that might not been candidated for it routinely. It is a serious ethical concern. Please explain.

*The vitreous fluids evaluated in this study were obtained from the cases with marked vitreous haze, whose diagnosis had been established by the meticulous observations of ocular involvement, clinical course, and concomitant systemic diseases combined with laboratory analyses. These patients had needed the vitreous biopsy to differentiate masquerade syndrome such as vitreoretinal lymphoma. Therefore, we have added the following sentences to the manuscript.*

*“All patients with idiopathic uveitis had marked vitreous haze, and underwent medically required vitrectomy because they were suspected masquerade syndrome such as vitreoretinal lymphoma.” (Page4, line 63-65)*
